# Correction: Towards control of excitonic coupling in DNA-templated Cy5 aggregates: the principal role of chemical substituent hydrophobicity and steric interactions

**DOI:** 10.1039/d4nr90140a

**Published:** 2024-07-24

**Authors:** Sebastián A. Díaz, Gissela Pascual, Lance K. Patten, Simon K. Roy, Adam Meares, Matthew Chiriboga, Kimihiro Susumu, William B. Knowlton, Paul D. Cunningham, Divita Mathur, Bernard Yurke, Igor L. Medintz, Jeunghoon Lee, Joseph S. Melinger

**Affiliations:** a Center for Bio/Molecular Science and Engineering Code 6900, U.S. Naval Research Laboratory Washington D.C. 20375 USA sebastian.diaz@nrl.navy.mil; b Micron School of Materials Science & Engineering, Boise State University Boise Idaho 83725 USA jeunghoonlee@boisestate.edu; c Volgenau School of Engineering, George Mason University Fairfax Virginia 22030 USA; d Optical Sciences Division, Code 5600, U.S. Naval Research Laboratory Washington DC USA; e Jacobs Corporation Hanover MD USA; f Department of Electrical & Computer Engineering, Boise State University Boise Idaho 83725 USA; g Electronics Science and Technology Division Code 6800, U.S. Naval Research Laboratory Washington D.C. 20375 USA joseph.melinger@nrl.navy.mil; h Department of Chemistry, Case Western Reserve University Cleveland OH 44106 USA; i Department of Chemistry & Biochemistry, Boise State University Boise Idaho 83725 USA

## Abstract

Correction for ‘Towards control of excitonic coupling in DNA-templated Cy5 aggregates: the principal role of chemical substituent hydrophobicity and steric interactions’ by Sebastián A. Díaz *et al.*, *Nanoscale*, 2023, **15**, 3284–3299. https://doi.org/10.1039/D2NR05544A.

The authors regret that an error occurred in the labelling of Fig. 3E, where the circular dichroism spectrum for Cy5-*t*Bu was mislabelled as Cy5-Cl and *vice versa*. This error strictly concerns the labelling of the figure and does not impact any of the data or conclusions of the article. The correct Fig. 3 is shown here.

**Fig. 1:**
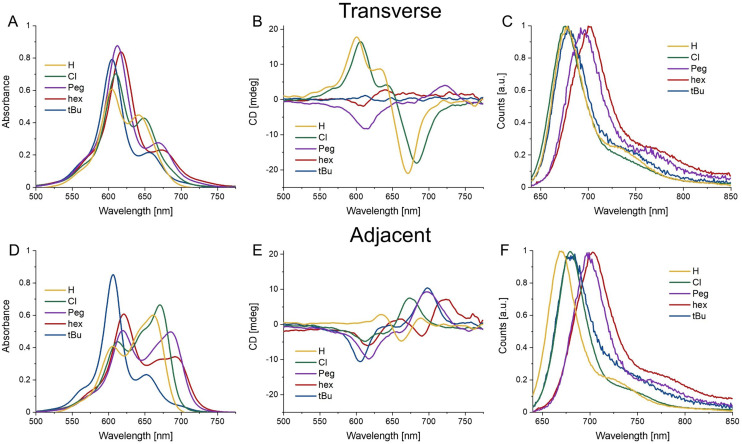
Steady state spectra of average HJ homodimers. (A–C) Absorbance, CD, and normalized emission spectra (excitation 615 nm) of HJ transverse homodimers of Cy5-R. (D–F) Absorbance, CD, and normalized emission spectra (excitation 615 nm) of HJ adjacent homodimers of Cy5-R. All measurements were performed at 20 °C.

The Royal Society of Chemistry apologises for these errors and any consequent inconvenience to authors and readers.

